# Separating the Wheat from the Chaff: The Use of Upstream Regulator Analysis to Identify True Differential Expression of Single Genes within Transcriptomic Datasets

**DOI:** 10.3390/ijms22126295

**Published:** 2021-06-11

**Authors:** Jeremiah Hadwen, Sarah Schock, Faraz Farooq, Alex MacKenzie, Julio Plaza-Diaz

**Affiliations:** 1 Department of Cellular and Molecular Medicine, Faculty of Medicine, University of Ottawa, Ottawa, ON K1H8M5, Canada; sarah.schock@canada.ca (S.S.); faraztfarooq@gmail.com (F.F.); 2 Children’s Hospital of Eastern Ontario Research Institute, Ottawa, ON K1H8L1, Canada; jrplaza@ugr.es; 3 Department of Biochemistry and Molecular Biology II, School of Pharmacy, University of Granada, 18071 Granada, Spain; 4 Instituto de Investigación Biosanitaria IBS.GRANADA, Complejo Hospitalario Universitario de Granada, 18014 Granada, Spain

**Keywords:** transcriptomics, differential expression analysis, rare disease, drug repurposing

## Abstract

The development of DNA microarray and RNA-sequencing technology has led to an explosion in the generation of transcriptomic differential expression data under a wide range of biologic systems including those recapitulating the monogenic muscular dystrophies. Data generation has increased exponentially due in large part to new platforms, improved cost-effectiveness, and processing speed. However, reproducibility and thus reliability of data remain a central issue, particularly when resource constraints limit experiments to single replicates. This was observed firsthand in a recent rare disease drug repurposing project involving RNA-seq-based transcriptomic profiling of primary cerebrocortical cultures incubated with clinic-ready blood–brain penetrant drugs. Given the low validation rates obtained for single differential expression genes, alternative approaches to identify with greater confidence genes that were truly differentially expressed in our dataset were explored. Here we outline a method for differential expression data analysis in the context of drug repurposing for rare diseases that incorporates the statistical rigour of the multigene analysis to bring greater predictive power in assessing individual gene modulation. Ingenuity Pathway Analysis upstream regulator analysis was applied to the differentially expressed genes from the Care4Rare Neuron Drug Screen transcriptomic database to identify three distinct signaling networks each perturbed by a different drug and involving a central upstream modulating protein: levothyroxine (*DIO3*), hydroxyurea (*FOXM1*), dexamethasone (*PPARD*). Differential expression of upstream regulator network related genes was next assessed in in vitro and in vivo systems by qPCR, revealing 5× and 10× increases in validation rates, respectively, when compared with our previous experience with individual genes in the dataset not associated with a network. The Ingenuity Pathway Analysis based gene prioritization may increase the predictive value of drug–gene interactions, especially in the context of assessing single-gene modulation in single-replicate experiments.

## 1. Introduction

Rare diseases including the muscular dystrophies system are significant contributors to human disability and illness. Although defined as less than 1 in 2000 people in Europe and less than 1 in 200,000 people in the United States, they are collectively common, carrying significant medical, societal, and economic costs [1,2,3,4].

There are comparatively few effective rare disease therapies; one approach to identifying new treatments involves genome-wide differential expression (DE) analysis (the process through which transcriptomic data are converted to statistical measures of gene expression) by microarray and RNA-sequencing [5,6,7,8,9,10,11,12], as a means of potentially correcting the pathogenic protein expression which underlies so many diseases [13].

Although the DE pipeline has been extensively customized for individual research purposes [14], the diverse approaches generally share the same workflow [15]: raw data processing, DE analysis, and multiple testing correction [16,17], followed by choice of a statistical cut-off to identify differentially expressed genes (DEGs). The DEGs must then be classified and validated to extract biologically useful information. Research involving transcriptome-wide DE data analysis may either focus on gene enrichment patterns or DE of single genes, this is particularly so when studying rare diseases for it is the modulation of a specific gene that can hold the greatest therapeutic promise (e.g., utrophin for Duchenne muscular dystrophy). In the former analysis, which relies primarily on the gene annotation and enrichment analysis [18], a certain degree of statistical rigour can be achieved by virtue of the number of differentially expressed genes being studied. One such example is the analysis of drug-mediated transcriptome modulation to identify novel indications for established drugs, pioneered by Lamb et al. who first used connectivity mapping of transcriptomic signatures to pair clinically approved drugs with disease signatures [19].

In contrast, single-gene modulation observed in system-wide datasets is not as reproducible, and this is especially the case in single-replicate experiments. This can be seen in efforts to repurpose drugs to treat rare monogenic diseases by mining transcriptome-wide drug screen data for drug hits on rare disease genes of interest [12,20,21,22,23]. This work, unlike the broader assessment of groups of genes outlined above, focuses on single-gene DE resulting in suboptimal reproducibility, particularly in single-replicate experimental systems. As a result, although several genes from these studies have been validated at the in vitro level and in whole-animal experiments, the validation rates were only 20% in vitro and 5% in vivo [12,20].

DEGs are often analyzed in aggregate using gene enrichment bioinformatic tools [24] or, more recently, commercial pathway analysis tools such as Ingenuity Pathway Analysis (IPA) [25]. IPA and similar tools (e.g., iPathwayGuide [26], PathVisio [27], GeneGO MetaCore [28]) are similar to gene-set enrichment analysis platforms [29], with the added benefit of knowing the direction (up- or downregulation) of each pathway. Based on annotated knowledge of functionally related DEGs, IPA upstream regulator analysis (URA) identifies a common upstream regulator (e.g., transcription factor, kinase, drug) and analyzes the DE of downstream genes. The tool then generates an associated z-score and *p*-value of overlap that impart statistical significance and directionality (up- or downregulation) to each upstream regulator [25].

Here, by marrying the statistical robustness of gene network analysis to single-gene DE measurement, we propose a method of gauging the validity of single DEGs identified from in vitro transcriptomic data, helping identify those that warrant further investigation in cellular and animal systems. Data from the Neuron Screen Database (available at http://bigbear.med.uottawa.ca:1000, accessed on 7 June 2021) [12] were analyzed by IPA URA to identify three DEG groups that share statistically activated or inhibited upstream regulators: (i) thyroid hormone upregulation of deiodinases (DIO) network as positive control for the URA methodology, (ii) novel hydroxyurea (HU)-mediated inhibition of *FOXM1* to investigate in vitro validation rates, and (iii) dexamethasone (DEX)-mediated activation of *PPARD* to investigate in vivo validation rates. The use of the URA-based method to select DEGs resulted in a validation rate far superior to that achieved previously for single genes not related to a network. This suggests the utility of the IPA-directed approach in distinguishing true DEGs from stochastic artifacts.

## 2. Results

### Pathway Analysis

URA of RNA-seq DE data from the recently published Neuron Screen of clinic-ready drugs [12] was conducted to assess whether an a priori “reliability” of an observed drug-conferred single-replicate single-gene DE might be achieved. Samples of “activated” and “inhibited” networks elicited by the 50 drugs that modulated the greatest number of transcripts were identified by IPA using upstream regulator analysis of DE data. Six statistically significant activated and inhibited URA networks (|z| > 2, and a minimum of five downstream DEGs with *p*-adj < 0.05) were selected for further study: Levothyroxine (DIO3), hydroxyurea (*FOXM1*), DEX (*PPARD*), DEX (*STAT4*), vigabatrin (*MKNK1*), and pregabalin (*PGR*) (Table 1).

**Figure 1:**
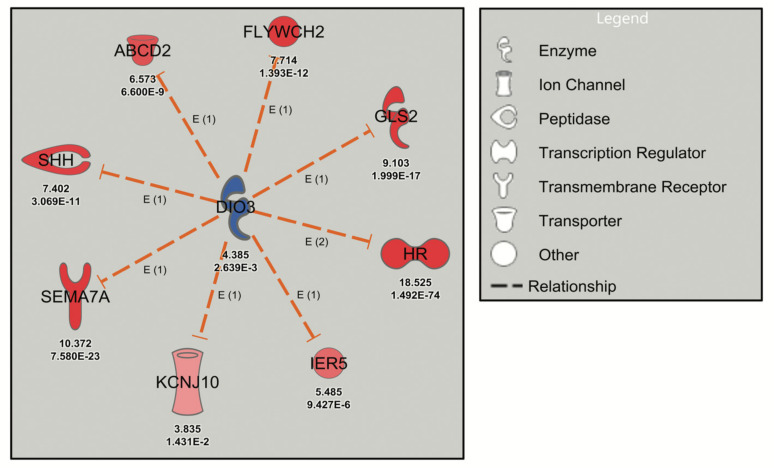
Robust URA network induced by levothyroxine treatment of mouse cerebrocortical cultures. Dashed orange lines signify activation (loss of inhibition) and red symbols signify upregulated genes with the Neuron Screen Z-score and adjusted *p*-value (*p*-adj) (<0.05) appearing directly below each symbol. The upstream regulator *DIO3* is blue, indicating downregulation based on the C4R drug screen data.

The deiodinase 3 (*DIO3*) network, inhibited by the thyroid hormone analog levothyroxine (T4) in the Neuron Screen data (z-score = −2.975), was initially analyzed. The *DIO3* gene, known to be regulated by thyroid hormone, encodes type 3 iodothyronine deiodinase that inactivates thyroid hormone and downregulates thyroid hormone-responsive genes [30]. The postulated downstream targets of *DIO3* (Figure 1) are known transcriptional targets of thyroid hormone (e.g., *Shh*, *Sema7a*, *Hr*) and have been validated in mouse cerebrocortex and cortical cultures [12,31]. Thus, the *DIO3* network was labelled with a z-score of −2.975, since the IPA software assumed inhibition of *DIO3* (although *DIO3* was in fact upregulated) because of the upregulation of thyroid hormone-responsive genes (Figure 1). This analysis serves to highlight the possible discrepancy that exists between an upstream regulator and the downstream genes, while confirming the reliability of URA network-directed prioritization of DEGs since all the genes in the *DIO3* network are upregulated in response to thyroid hormone experimentally (from the Neuron Screen data) and in the published literature. Another example is the *STAT4* network, which has a robust activation z-score with 12 network-related genes. This occurs despite the fact that *STAT4* did not appear as a differentially expressed gene in the Neuron Screen data, and thus there is no fold expression present. This then is an example of using this approach to identify a potential upstream regulator in the absence of it showing up as a DEG itself.

**Figure 2:**
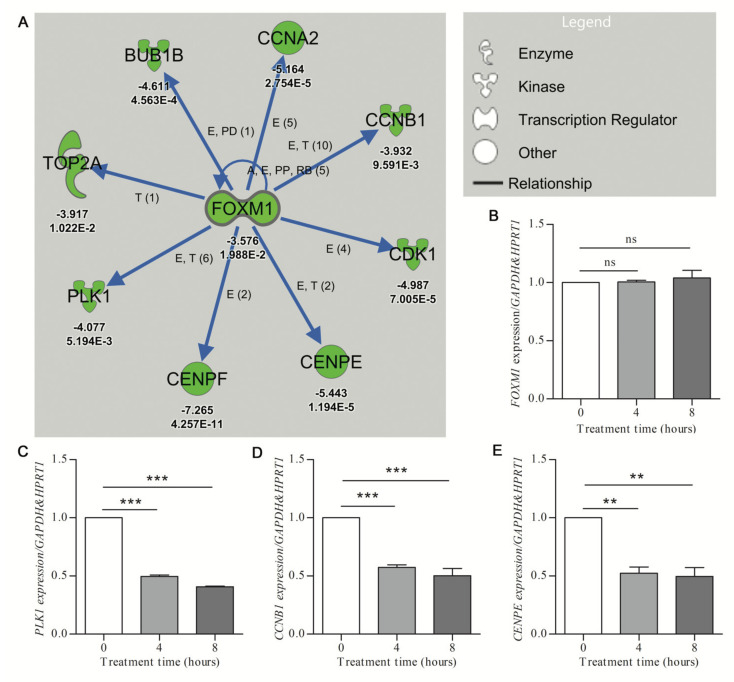
Validation of downregulated gene expression prioritized by IPA analysis. (**A**) The URA network shows downregulation (green symbols) of the putative upstream regulator (*FOXM1*) and the downstream molecules (with associated Z-score and *p*-value given under each gene, for *p* values, E-n symbolizes E^-n^)) (Care4Rare Neuron Screen). Blue arrows indicate inhibition of the physiological activating relationship between *FOXM1* and related genes. Only genes for which *p*-adj < 0.05 are included. (**B–E**) U87 glioblastoma cells were treated with 250 μM hydroxyurea for 0, 4, and 8 h. qRT-PCR (*n* = 3) was employed to determine target gene expression with geometric normalization against *GAPDH* and *HPRT1*. (**B**) Fork-head box M1 (*FOXM1*). (**C**) Polo-like kinase 1 (*PLK1*). (**D**) Cyclin B1 (*CCNB1*). (**E**) Centromere protein E (*CENPE*). Statistical significance was measured by one-way ANOVA (nonparametric) with Tukey post hoc analysis (** *p* < 0.01, *** *p* < 0.001, ns = nonsignificant).

The *FOXM1* network, inhibited by treatment with hydroxyurea (HU) (activation z-score of −3.245; *p*-value of overlap at 1.63 × 10^−10^) was used to determine the rate of in vitro validation of network-associated DEGs. The *FOXM1* transcription factor has documented roles both in cell cycle gene regulation and DNA damage repair cascade activation [32]. However, the role of HU in the transcriptional modulation of the *FOXM1* network genes has not been well described. The IPA analysis showed that eight *FOXM1*-target genes involved in G2 (growth phase 2) and M phase (mitosis phase) of cell cycle progression were downregulated in response to hydroxyurea treatment or mouse cortical cultures (Figure 2A). To determine the validation rate of the HU-elicited *FOXM1*-associated DEGs, human U87 glioblastoma cells were treated for 0, 4, and 8 h with 250 μM HU. Quantitative RT-PCR revealed HU-mediated increases (minimum significance of *p* < 0.05) of seven of the eight *FOXM1*-network genes at 4 and/or 8 h (Figure 2C–E; Supplementary File, Figure S1). The level of the upstream regulator gene *FOXM1* was not significantly affected by 4 or 8 h of HU treatment (Figure 2B). These results confirmed the efficiency of identifying DE genes from URA network analysis, while serving to highlight that such results are not contingent on the individual upstream gene (in this case *FOXM1*).

**Figure 3:**
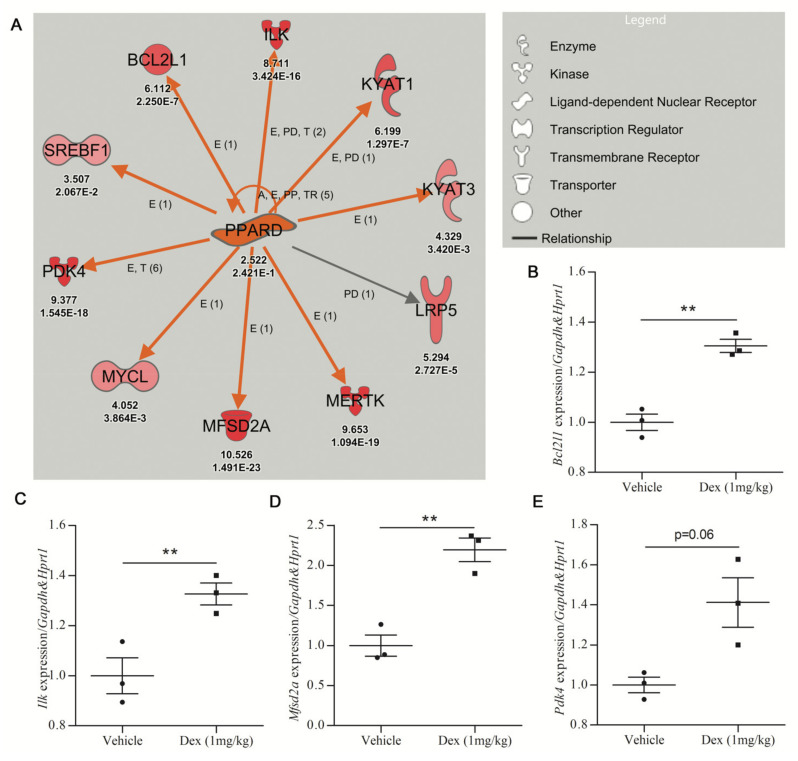
Validation efficiency for upregulated gene expression prioritized by IPA analysis. (**A**) The *PPARD* upstream regulator identified by URA analysis is upregulated in dexamethasone-treated mouse cerebrocortical cultures (Care4Rare Neuron Screen). Orange arrows signify an activating relationship with downstream genes and red symbols signify upregulated genes with the Neuron Screen Z-score and *p*-adj appearing directly below each symbol. Only genes for which *p*-adj < 0.05 are included. (**B–E**) Adult male C57BL6 mice were treated p.o. with vehicle or DEX (1 mg/kg) for 5 days, and qRT-PCR was employed to determine gene expression of 7 of the 10 targets of *PPARD* (*n* = 3). The four genes with the most robust upregulation are shown. (**B**) Bcl-2-like protein 1 (*Bcldl1*). (**C**) Integrin-linked kinase (*Ilk*). (**D**) Major facilitator superfamily domain containing 2A (*Mfsd2a*). (**E**) Pyruvate dehydrogenase kinase 4 (*Pdk4*). For all hits, statistical significance was measured by Student’s paired two-tailed *t*-test (** *p* < 0.01).

The *PPARD* network, activated by DEX treatment (z-score = 3.126 and *p*-value = 0.0458), was next used to assess the in vivo validation of DEGs identified by URA networks. The activity of 1 mg/kg DEX oral treatments was confirmed by the upregulation of the DEX-responsive gene *Fkbp5* in the cortex of C57BL6 mice shown by qRT-PCR [33,34] (Supplementary File, Figure S2A). Subsequently, qRT-PCR analysis of the *PPARD* network in cortical tissue from DEX-treated mice was undertaken. Seven of the ten genes in the *PPARD* network were studied by qRT-PCR. Three genes (*Bcl2l1*, *Ilk*, *Mfsd2a*) showed statistically significant upregulation in DEX-treated mice (Figure 3B–D), while *Pdk4* and *Kyat3* showed a trend toward upregulation in DEX-treated mice (Figure 3E; Supplementary File, Figure S2B). Only two of the seven genes tested (*Mertk* and *Lrp5*) showed no increase in response to DEX treatment in the mouse cortex (Supplementary File, Figure S2C,D).

## 3. Discussion

Recent technologies have permitted the massively parallel interrogation of biologic systems generating many orders of magnitude of data points. The challenge is how to extract reliable information of biological utility from such massive sequencing and gene-array datasets. In this regard, although multiple biological replicates are necessary for traditional statistical calculations, it is not always feasible (high cost of RNA-seq) or possible (personalized medicine) to use biological replicates to truly validate a given result.

This problem is offset to some extent when a subset of data is analyzed, as the statistical strength of such profiles is greater than that achieved with the measurement of a single data point. This can be seen in rare disease drug repurposing studies utilizing genome-wide DE analyses that involve the orthogonal signature technique, pioneered by Lamb et al. (2006) [19,35,36]. In contrast, our lab has adopted a more targeted approach, mining DE datasets to identify drug-based modulation of single genes with potential therapeutic benefit for monogenic diseases [12,20,21,22]. However, the inherent variability of single-gene instances within system-wide datasets is borne out by in vitro and in vivo transcriptional validation rates of 20% or lower documented in these studies.

In particular, in the analysis of our recent RNA-seq drug screen using primary cerebrocortical cultures, the attempted validation of 32 DEGs taken from 60 rare neurogenetic disease-associated genes originally catalogued by Mears et al. (2017) was successful in only six cases, a validation rate of less than 20% [12,20].

One technique that has been used to solve the lack of biological replicates is hypergeometric distribution analysis, which has been proposed for individualized therapy development for glioblastoma multiforme [9]. In an alternative approach, we have in the present study explored the use of IPA-based URA to assign a reliability probability for single-gene instances found within system-wide RNA-seq datasets. The URA technique is well suited given the lack of replicates, as the addition of a minimum of four biologically related genes to serve as biological replicates of a gene of interest dramatically increases the efficiency of the individual gene validation. Importantly, our technique of reliably identifying DEGs does not depend on the degree of gene expression of the upstream regulator. For example, validation of the *FOXM1* network revealed a high rate of validation of downstream genes (e.g., *PLK1*, *CCNB1*), yet a lack of validation of the *FOXM1* gene itself (despite being downregulated in the RNA-seq data in response to HU, it was not downregulated in qRT-PCR validation). With regard to the STAT4 network, gene expression results for *STAT4* were not even included in the original RNA-seq data (that were input into the IPA software). However, the network achieved statistical significance due to the number of transcriptional targets of STAT4 that were upregulated by DEX.

Our robust networks include a minimum of five DEGs per URA network both for optimal statistical strength and given that roughly a fourth of protein-coding genes have been linked to a rare genetic disease [37,38] (with the general expectation that this proportion will increase with the ongoing delineation of novel ultrarare diseases). Each network would thus have a reasonable probability of including at least one rare disease gene; for example, rare disease-causing genes are seen in both the HU-responsive *FOXM1* network (*CENPE*, *CENPF*) [39,40] and the DEX-responsive PPARD network (*MERTK*, *MFSD2A*) [41,42]. Obviously, a robust network could comprise a different number of genes depending on the research question being posed.

Several other studies using URA-based analysis of networks to interpret transcriptomic data, while not quantifying the validation rate, have reported good reproducibility [43,44,45,46]. However, in contrast to the present report, the goal of these studies was to validate the upstream regulators, not to identify and prioritize DEGs for validation as we have done. Although the sample size was small, our network-directed prioritization of DEGs resulted in 100% validation of mouse cerebrocortical culture hits in human immortalized cultures (*FOXM1* DEGs). Moreover, a roughly 40% validation from tissue culture to whole animals was documented (*PPARD* DEGs). Collectively, the results suggest that for drug-conferred DEGs that are also constituents of a robust transcriptional network response, there is a greater likelihood of validation in the original system as well as in other in vitro and in vivo models, even extending to different species, thus representing true physiological relationships that transcend the boundaries of species and experimental conditions.

Finally, novel network analyses, such as weighted correlation network analysis (WGCNA), could be used to search for clusters of highly correlated genes [47]. WGCNA algorithms have been used in dyslipidemias [48], cancer [49,50], and autism spectrum disorder [51].

## 4. Materials and Methods

### 4.1. Animals and Treatments

All animal protocols were approved by the Animal Care and Veterinary Services (ACVS) and Ethics Board of the University of Ottawa. For in vivo drug studies, male C57BL6 mice (8 weeks old) were obtained from Charles River, housed in triplicate, and given food and water *ad libitum*. Mice were treated once daily with 1 mg/kg DEX (Sigma-Aldrich, Oakville, ON, Canada) by oral gavage for 5 days. Roughly four hours after the last dose, the mice were anesthetized by isoflurane (Sigma-Aldrich, ON, Canada) and euthanized by cervical dislocation. Cerebral cortices were then collected from each animal and flash-frozen in liquid nitrogen. Total RNA extraction from the cortex was performed by QIAzol lysis reagent according to the manufacturer’s recommendations (Qiagen, Montreal, QC, Canada). The RNA was then purified over Qiagen RNeasy Mini spin columns (Qiagen, Montreal, QC, Canada) and frozen at −80 °C.

### 4.2. Human Glioblastoma Cell Culture

Human U87 glioblastoma cells (ATCC) were cultured in DMEM high-glucose supplemented with 10% fetal calf serum (Life Technologies, Burlington, ON, Canada) and 2 mM L-glutamine (Life Technologies, ON, Canada). For the validation of *FOXM1* targets, U87 cells were plated in 10 cm dishes at 120,000 cells per dish in a volume of 10 mL. After an overnight settling period, U87 cells were treated for 0, 4, or 8 h with 0.25 mM HU (Sigma-Aldrich, ON, Canada). After treatment end-points, cells were washed with 1 × phosphate-buffered saline (PBS) (Sigma-Aldrich, ON, Canada), trypsinized, and pelleted by centrifugation at 300× *g* for 5 min. Pellets were rinsed in 10 mL of 1 × PBS and then stored at −8 °C. RNA extraction was performed by Qiagen RNeasy Mini column-based extraction (Qiagen, Montreal, QC, Canada) and purification (Qiagen, Montreal, QC, Canada) according to the manufacturer’s instructions.

### 4.3. Dataset Used and Ingenuity Pathway Analysis

Primary murine neuronal cultures were incubated with 219 drugs mostly derived from the Screen-Well Food and Drug Administration approved drug library v2 (Enzo Life Sciences). Drugs were selected for blood–brain barrier penetrance, comparative safety consistent with long-term use, and oral bioavailability representing 80 therapeutic classes, using concentrations approximating those attained in patients (more details are available at http://bigbear.med.uottawa.ca:1000/, accessed on 7 June 2021). cDNA libraries, constructed from the poly-A fraction of total RNA extracted from drug- and control-treated cultures, were sequenced and analyzed, establishing a transcriptome-wide DE dataset representing all 227 sequenced libraries. For each sample, gene expression for 14,000 genes with cpm > 1 was normalized against the average of all conditions. Given that the screen was performed as a single replicate, the expression of a given gene across all samples served as the background to represent unaffected expression distributions. Robust z-scores and *p*-adj for each potential drug–gene interaction were generated, with DE being defined as *p*-adj < 0.05. The transcriptome-wide DE datasets of 50 of the most transcriptionally active drugs from the Neuron Screen were entered into the IPA software. Default settings were used for the analysis with a statistical limit of z-score > 3 for all data points. The two metrics of differential expression that were entered were “z-score” and *p*-adj. Each dataset was individually analyzed by IPA, and the URA function was used to identify upstream regulator pathways that were “activated” (z-score > 2) or “inhibited” (z-score < −2). IPA also calculated the *p*-value of overlap that was considered significant if *p* < 0.05. Upstream regulator networks chosen for validation were curated by removing genes that had *p*-adj > 0.05. Networks identified as “robust” contained ≥ 5 statistically significant gene-targets that were modulated in a direction consistent with the IPA prediction. 

### 4.4. Quantitative qRT-PCR

DEGs from robust URA networks were investigated by qPCR in human U87 cells and C57BL6 mouse cortex. Gene-specific primers were designed for each gene of interest, and *Gapdh* and *Hprt1* were used as housekeeping genes (Table S1). Primers for qPCR were designed using NCBI Primer-BLAST in accordance with the MIQE guidelines. Reverse transcription of purified RNA using the Bio-Rad iScript advanced RT kit (Bio-rad, Mississauga, ON, Canada) on a T100 Thermal Cycler (Bio-rad, Mississauga, ON, Canada) and qPCR using the iQ SYBR Green Master Mix (Bio-Rad, Mississauga, ON, Canada) on a CFX96 Touch Real-Time PCR Detection System (Bio-rad, Mississauga, ON, Canada) were performed as previously described [12]. Bio-Rad CFX software (Bio-rad, Mississauga, ON, Canada) enabled geometric mean normalization of target gene expression to *Gapdh* and *Hprt1*.

### 4.5. Statistical Analysis

Statistical tests were performed using IBM SPSS Statistics for Windows, Version 25.0 (IBM Corp., Armonk, NY, USA). All data are expressed as mean and standard deviation unless otherwise indicated. Statistical significance was measured by one-way ANOVA with Tukey post hoc analysis (for in vitro validation) and by Student’s paired two-tailed *t*-test (for in vivo validation).

## 5. Conclusions

In conclusion, we present a method to prioritize the investigation of DEGs identified in transcriptome-wide studies in neurogenetic disease. We believe our approach, combining the statistical rigour of gene enrichment and pathway analysis while utilizing accessible bioinformatics tools, may serve as a useful low-cost rapid filter to prioritize single DEGs worthy of further analysis. Although IPA was used for gene prioritization in our study, it is likely that other roughly equivalent tools (iPathwayGuide, PathVisio, GeneGo) would allow for similar gene prioritization. As an ever-expanding number of organisms are being added to the list of sequenced genomes, pathway tools such as IPA may be developed that can integrate gene expression results of nonmodel organisms. The URA-directed prioritization technique could then be broadly applicable in the context of transcriptomic data with impacts in fields as diverse as personalized drug discovery, the effect of ocean acidification on marine species, or elucidating mechanisms of antibiotic resistance.

## Figures and Tables

**Table 1:** Summary of six upstream regulators identified in the Neuron Screen data.

Drug Name	Upstream Regulator	Expression Fold Change	Predicted Activation State	Activation Z-Score	*p*-Value of Overlap	Number of Genes
Levothyroxine	DIO3	4.385	Inhibited	−2.975	1.79 × 10^−7^	8
Hydroxyurea	FOXM1	−3.579	Inhibited	−3.245	1.63 × 10^−10^	8
Dexamethasone	PPARD	2.522	Activated	3.126	4.58 × 10^−2^	10
Dexamethasone	STAT4	NA	Activated	2.933	7.36 × 10^−4^	12
Vigabatrin	MKNK1	1.187	Inhibited	−3	1.40 × 10^−4^	5
Pregabalin	PGR	1.013	Activated	3.376	7.77 × 10^−9^	13

The expression fold change indicated is derived from the original study reporting the RNA-seq results from the neuronal screen. The level of mRNA (i.e., RNA-seq read numbers) for a given gene in the presence of a drug is compared against the average mRNA level of that gene for all conditions while the upstream regulator is the central molecule in the network. The activation z-score serves as a statistical measure as well as a directional tool (negative/downregulated, positive/upregulated) while the *p*-value of overlap serves as an additional measure of statistical certainty pertaining to the interrelated nature of the molecules forming each network. NA, data not available as gene expression was not included in the Neuron Screen data [25].

## Data Availability

The Care4Rare Neuron Screen database that was used to identify the transcriptional drug–gene hits can be accessed from http://bigbear.med.uottawa.ca:1000, accessed on 7 June 2021. The original fastq files generated and analyzed to create the database are available from the Gene Expression Omnibus (GEO) database (https://www.ncbi.nlm.nih.gov/geo/) (accession number GSE110256, accessed on 7 June 2021).

## Supplementary Materials

The following are available online at https://www.mdpi.com/article/10.3390/ijms22126295/s1, Figure S1: Validation of the effect of HU on four *FOXM1* downstream genes. U87 glioblastoma cells were treated for 0, 4, and 8 h, and qRT-PCR was employed to determine gene expression of (A) cyclin-dependent kinase 1 (*CDK1*), (B) centromere protein F (*CENPF*), (C) BUB1 mitotic checkpoint serine/threonine kinase (*BUB1*), and (D) cyclin A2 (*CCNA2*). Statistical significance was measured by one-way ANOVA (nonparametric) with Tukey post hoc analysis (** *p* < 0.01, *** *p* < 0.001, ns = not significant), Figure S2: Validation of the effect of dexamethasone on three *PPARD* targets in vivo. Male C57BL6 mice were treated orally for 5 days with vehicle (*n* = 3) or DEX (1 mg/kg) (*n* = 3). Gene expression of *PPARD* targets was determined by qRT-PCR. (A) FKBP prolyl isomerase 5 (*Fkbp5*), (B) kynurenine aminotransferase 3 (*Kyat3*), (C) C-mer proto-oncogene tyrosine kinase (*Mertk*), (D) LDL receptor-related protein 5 (*Lrp5*), Table S1: Mouse and human primers.
