# Supplementary material for: Separating the Wheat from the Chaff: The Use of Upstream Regulator Analysis to Identify True Differential Expression of Single Genes within Transcriptomic Datasets
Source: Int J Mol Sci. 2021 Jun 11;22(12):6295. doi: 10.3390/ijms22126295 (PMC8231191; doi:10.3390/ijms22126295)
Supplement: Supplementary file 1 [file ijms-22-06295-s001.zip › ijms-1228153-supplementary.pdf]

International Journal of
Molecular Sciences

MDPI

Supplementary Material

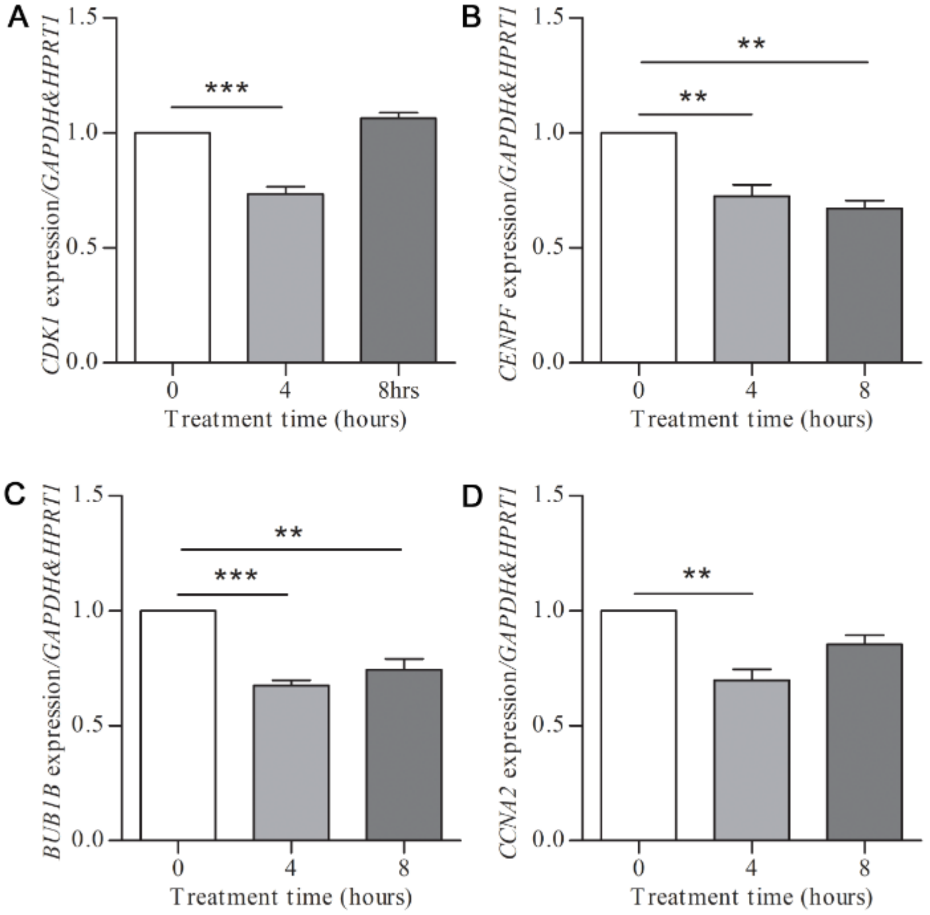

Figure S1. Validation of the effect of HU on four FOXM1 downstream genes. U87 glioblastoma cells were treated for 0, 4, and 8 h, and qRT-PCR was employed to determine gene expression of (**A**) cyclin-dependent kinase 1 (CDK1), (**B**) centromere protein F (CENPF), (**C**) BUB1 mitotic checkpoint serine/threonine kinase (BUB1), and (**D**) cyclin A2 (CCNA2). Statistical significance was measured by one-way ANOVA (nonparametric) with Tukey post hoc analysis (** $p < 0.01$, *** $p < 0.001$, ns = not significant).

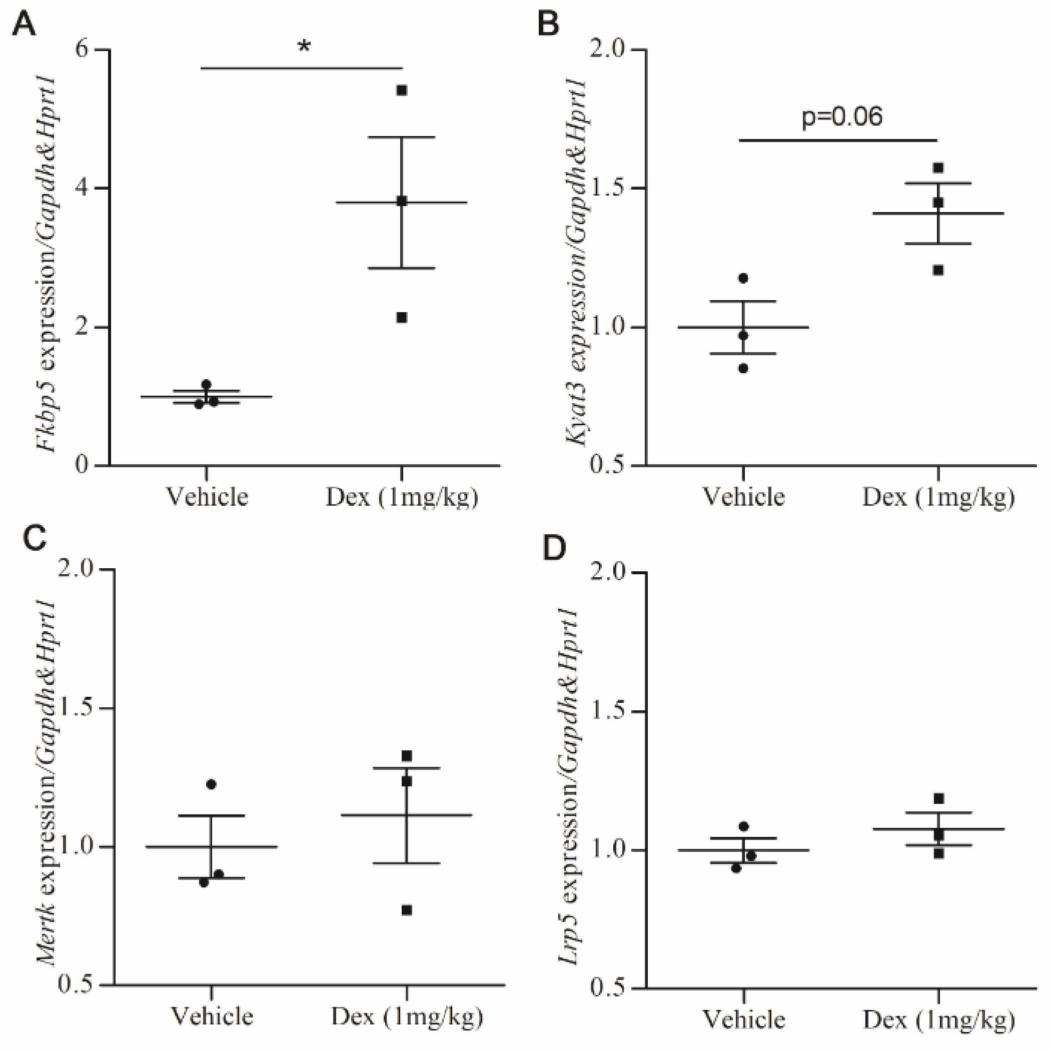

Figure S2. Validation of the effect of dexamethasone on three PPARD targets in vivo. Male C57BL6 mice were treated orally for 5 days with vehicle (n = 3) or DEX (1 mg/kg) (n = 3). Gene expression of PPARD targets was determined by qRT-PCR. (**A**) FKBP prolyl isomerase 5 (Fkbp5), (**B**) kynurenine aminotransferase 3 (Kyat3), (**C**) C-mer proto-oncogene tyrosine kinase (Mertk), (**D**) LDL receptor-related protein 5 (Lrp5).

Table S1. Mouse and human primers.

Mouse Primers Employed for qPCR		
**Gene Symbol**	**Forward Primer**	**Reverse Primer**
Gapdh	CGTCCCGTAGACAAAATGGT	CTCCTGGAAGATGGTGATGG
Hprt1	GCAAACTTTGCATTCCCTGGTT	CAAGGGCATATCCAACAACA
Klf9	GGAAACACGCCTCCGAAAAG	AACGGAACTGCTTTTCCCCA
Fkbp5	CCATGACTGAGCAGGGTGAA	CCTCGTCACTAGTCCCCACT
Ilk	TGGACAACACAGAGAACGACC	GGGGTATCATCCCCACGATTC
Bcl2l1	CCTTGGATCCAGGAGAACGG	TCAGGAACCAGCGGTTGAAG
Pdk4	CGTACTCCACTGCTCCAACA	ACACCAGTCATCAGCTTCGG
Mfsd2a	CCTTCACTGACCCTCTGGTG	GAAGCCGTGTGAACTTTCCG
Mertk	ACGTTGGTGGATACGTGCAT	CTCTTCCCACTTCTCGGCAG
Lrp5	GCCTTCATGGATGGGACCAA	GCCCGTTCAATGCTATGCAG
Kyat3	GTCCTCGGACTCTGCACTTC	AGGATCCGCAGCCAACTTAG
**Human Primers Employed for qPCR**		
**Gene Symbol**	**Forward Primer**	**Reverse Primer**
GAPDH	GTTCGACAGTCAGCCGCATC	AGTTAAAAGCAGCCCTGGTGA
HPRT1	TGACACTGGCAAACCAATGCA	GGTCCTTTTCACCAGCAAGCT
BUB1B	CTTCTGGGATGGGTCCTTCTG	GCTCTGAGGCAGCAATCTGT
FOXM1	AGCGGCCACCCTACTCTTA	CCCTGGGTCCAGTGGCTTAAA
CCNA2	CGTGAAGATGCCCTGGCTTT	AACCAGTCCACGAGGATAGC
CENPF	CTGCGGGCAGTTTGAATTAG	CTCTTGTAGGCAGCCCTTCT
CENPE	TGAACTCACTTCGTGCTGACT	ACTTCTGCATGCTTAACTAAATTCT
PLK1	AGTGTCAATGCCTCCAAGCC	AGAGGATGAGGCGTGTTGAG
CCNB1	CCTCTCCAAGCCCAATGGAA	ACTTCCCGACCCAGTAGGTA
CDK1	CGCGGAATAATAAGCCGGGA	AGGAACCCCTTCCTCTTCACT
